# Development and validation of a preoperative clinical parameter-based nomogram to predict overt hepatic encephalopathy within 1 year after transjugular intrahepatic portosystemic shunt

**DOI:** 10.3389/fmed.2025.1634368

**Published:** 2025-10-20

**Authors:** Zhimeng Jiang, Jianguo Chu, Zheyi Han, Baojie Wei, Zhibo Xia, Tongzhen Zhang, Ying Zhu, Nianjun Xiao, Shoubin Ning

**Affiliations:** ^1^Graduate School of Hebei North University, Zhangjiakou, Hebei Province, China; ^2^Department of Gastroenterology, Air Force Medical Center, Chinese People's Liberation Army, Beijing, China

**Keywords:** transjugular intrahepatic portosystemic shunt, overt hepatic encephalopathy, cirrhosis, prognostic nutritional index, nomogram, predictive model

## Abstract

**Background and objective:**

Transjugular intrahepatic portosystemic shunt (TIPS) is an important intervention for relieving portal hypertension-related complications in patients with decompensated cirrhosis. However, over-hepatic encephalopathy (OHE) after TIPS is common and significantly impacts patients’ prognosis and quality of life. There is an urgent need for an effective predictive model to evaluate the risk of OHE. This study aims to develop and validate a practical, accessible, and high-performance predictive model for OHE based on preoperative clinical parameters.

**Methods:**

A total of 440 patients with decompensated cirrhosis who underwent their first TIPS procedure between January 2017 and December 2023 were retrospectively enrolled and randomly divided into training (*n* = 310) and validation (*n* = 130) cohorts in a 7:3 ratio. Least absolute shrinkage and selection operator (LASSO) regression was used for variable selection, followed by multivariate logistic regression to construct the predictive model, which was visualized as a nomogram. The model’s performance was evaluated using the area under the receiver operating characteristic curve (AUC), calibration curves, decision curve analysis (DCA), and clinical impact curves (CIC).

**Results:**

LASSO regression selected five predictors from 34 variables: prognostic nutritional index (PNI), age, previous history of hepatic encephalopathy, serum ammonia, and creatinine. The model achieved an AUC of 0.8835 (95% CI: 0.8408–0.9262) in the training cohort, outperforming MELD (AUC: 0.7204) and CTP scores (AUC: 0.6576). In the validation cohort, the AUC was 0.858, indicating good discrimination. Calibration curves, DCA, and CIC also demonstrated strong model accuracy and clinical utility.

**Conclusion:**

The prediction model based on preoperative clinical parameters accurately assesses the 1-year risk of OHE after TIPS in patients with cirrhosis and may serve as a practical tool for clinical decision-making.

## Introduction

Cirrhosis is a global public health issue. According to the World Health Organization, cirrhosis-related deaths account for approximately 2.4% of global mortality. In the decompensated stage, portal hypertension worsens and may lead to severe complications such as refractory ascites and variceal bleeding, with an annual mortality rate of 20–57% (1, 2). Transjugular intrahepatic portosystemic shunt (TIPS), a minimally invasive procedure, offers advantages such as low trauma, rapid recovery, and effective portal pressure reduction. It has become a first-line therapy for refractory ascites and secondary prevention of variceal bleeding (3).

However, the incidence of hepatic encephalopathy (HE) after TIPS remains high, with reported rates ranging from 23 to 54.5% (4, 5). The West Haven criteria categorize HE severity into covert HE (grades 0–1) and overt hepatic encephalopathy (OHE) (grades 2–4) (6, 7), with post-TIPS OHE incidence reaching 30–45% (8). This complication can necessitate shunt reduction or embolization, increasing economic burden and hospitalization time while impairing quality of life and long-term prognosis. It also complicates clinical decision-making regarding TIPS eligibility.

Clinical prediction models, which integrate clinical, laboratory, and imaging data, are increasingly used to predict disease progression and treatment response, supporting personalized and precision medicine (9). Although Child-Turcotte-Pugh (CTP) and Model for end-stage liver disease (MELD) scores are commonly used to quantify liver function and estimate prognosis, they were not specifically developed to predict post-TIPS OHE and have limitations in this setting—such as inclusion of subjective items (e.g., ascites, HE grade) and limited incorporation of nutritional and inflammatory markers that are increasingly recognized as relevant to HE pathogenesis.

Thus, this study aimed to build and validate a multivariate clinical prediction model using quantifiable preoperative indicators to estimate the 1-year risk of OHE in patients with decompensated cirrhosis after TIPS. The goal is to provide clinicians with an intuitive and practical preoperative tool for identifying high-risk patients and optimizing interventions to advance TIPS-based care.

## Methods

### Study population and grouping

**Figure 1:**
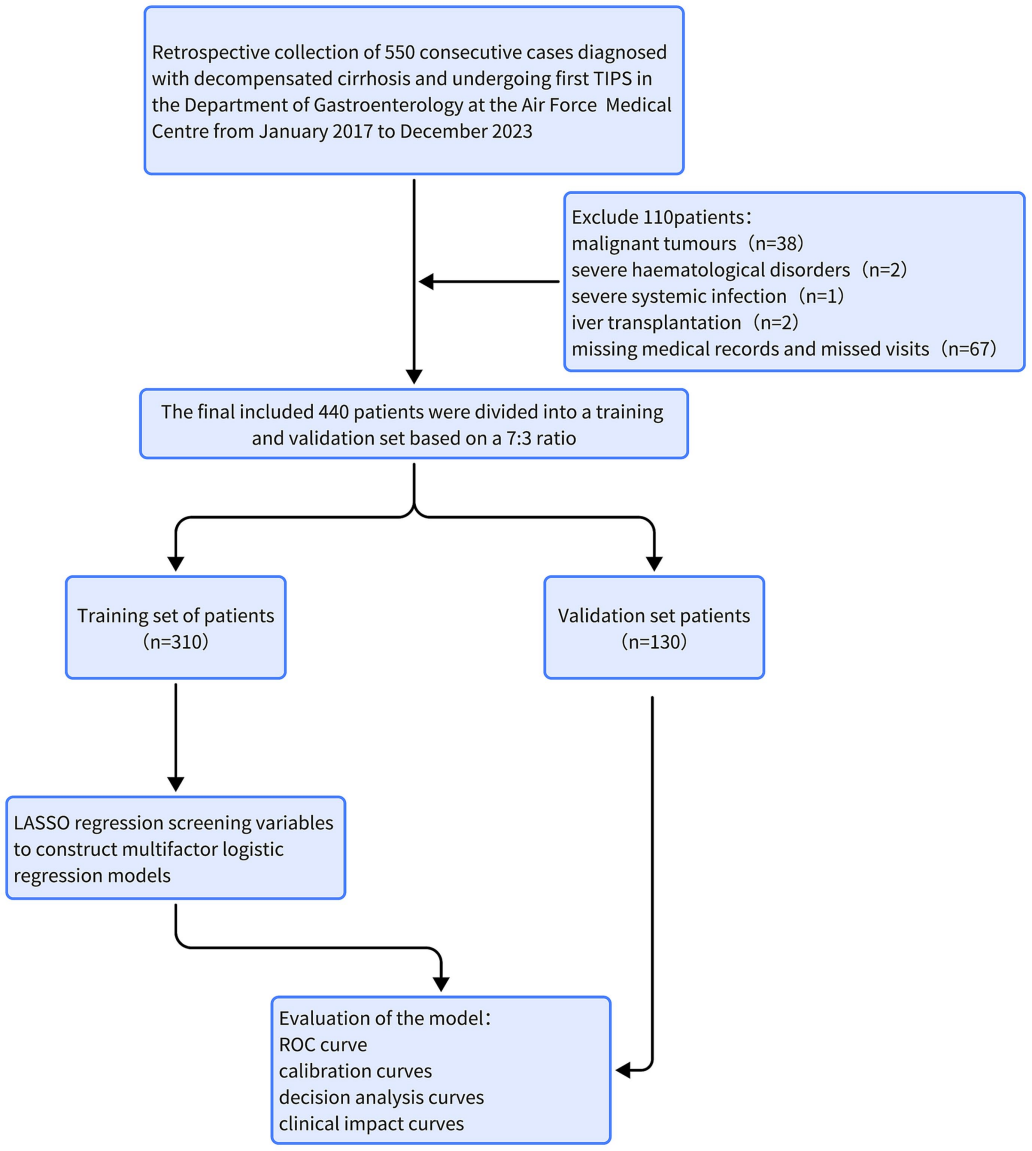
Flow chart of participants selection.

This single-center retrospective study included 550 patients with decompensated cirrhosis who underwent first-time TIPS between January 2017 and December 2023 at the Department of Gastroenterology, Air Force Medical Center. After excluding patients with malignancies (*n* = 38), severe hematologic disorders (*n* = 2), systemic infections (*n* = 1), post-transplantation (*n* = 2), and missing data or loss to follow-up (*n* = 67), 440 patients were enrolled. Based on OHE occurrence within 1-year post-TIPS, patients were categorized into OHE and non-OHE groups and randomly split into training (*n* = 310) and validation (*n* = 130) cohorts (Figure 1).

### Inclusion criteria

(1) Cirrhosis confirmed by pathology, imaging, or clinical criteria (10, 11); (2) First-time TIPS procedure; (3) Complete perioperative clinical data available.

### Exclusion criteria

(1) Malignancies; (2) Severe renal insufficiency, hematologic diseases, uncontrolled systemic infections; (3) History of liver transplantation; (4) Recent psychiatric medication use or neuropsychiatric disorders; (5) Lost to follow-up postoperatively.

### Data collection

Demographics (age, sex), lifestyle (smoking, alcohol), laboratory tests (liver/renal function, coagulation, lipids, white blood cell count, hemoglobin, body mass index, albumin/globulin ratio), and clinical history (diabetes, hypertension, cirrhosis etiology, TIPS indication, previous history of HE) were recorded. Prognostic nutritional index (PNI) = albumin (g/L) + 5 × lymphocyte count (10^9^/L) (12). CTP score: total bilirubin, albumin, prothrombin time, ascites, HE (13). MELD score = 11.2 × ln(International Normalized Ratio) + 9.57 × ln(creatinine, mg/dL) + 3.78 × ln(total bilirubin, mg/dL) + 6.43 (14).

### Outcome definition

The primary outcome was the occurrence of OHE (West Haven grade ≥2) within 1 year post-TIPS (6). Diagnosis was adjudicated by two independent hepatologists (blinded to model predictors) based on in-hospital medical records, outpatient evaluations, and structured telephone interviews during follow-up. The structured telephone questionnaire asked about specific symptoms/signs (e.g., confusion/disorientation, somnolence, asterixis, slurred speech, behavioral change). If telephone screening suggested possible OHE, investigators reviewed the hospital/emergency records and, when necessary, consulted an expert hepatologist; disagreements were resolved by a third senior hepatologist. The first OHE episode after TIPS during the 12-month follow-up was considered the study endpoint.

### Ethical approval

Approved by the Ethics Committee of the Air Force Medical Center. All participants signed informed consent (Air Force Special Forces (Research) No. 2025-38-PJ01). The study adhered to the Declaration of Helsinki.

### Statistical analysis

Analysis of variance and Chi-square tests were used to compare continuous and categorical variables. After random allocation to training and validation cohorts (7:3), to avoid overfitting and multicollinearity, variable selection using LASSO regression (10-fold cross-validation) was performed only within the training cohort t. Variables retained by LASSO were entered into multivariable logistic regression to derive the final model and to construct a nomogram. Model performance was evaluated in both the training and independent validation cohorts. Variables selected by LASSO were subjected to Spearman correlation and multivariate logistic regression to build the model and nomogram.

Model discrimination was assessed using the area under the receiver operating characteristic curve (AUC), calibration with calibration curves, and clinical utility with decision curve analysis (DCA), and clinical impact curves (CIC). All analyses were performed using R software (v4.1.3). A two-sided *p* < 0.05 was considered statistically significant.

## Results

### Baseline characteristics

Among 440 patients, the mean age was 54.55 ± 12.77 years; 284 were male (64.55%), 156 female (35.45%). TIPS indications included variceal bleeding (*n* = 271, 61.59%), refractory ascites (*n* = 139, 31.59%), and others (*n* = 30, 6.82%). Cirrhosis etiologies included hepatitis B (*n* = 205, 46.59%), alcohol (*n* = 64, 14.55%), autoimmune liver disease (*n* = 25, 5.68%), cavernous transformation of portal vein (*n* = 17, 3.86%), and others (*n* = 129, 29.32%). Compared to the non-OHE group, OHE patients had significantly higher age, ammonia, creatinine, uric acid, total bile acid, Prothrombin time, MELD, and CTP scores, and lower high density lipoprotein cholesterol, cholinesterase, PNI, and hemoglobin (*p* < 0.05) (Table 1).

**Table 1:** Baseline characteristics of the study population.

Characteristic	Total patients (*N* = 440)	Non-OHE (*N* = 338)	OHE (*N* = 102)	*p*-value
Serum ammonia (μmol/L)	62.63 ± 32.36	57.01 ± 29.98	81.26 ± 33.08	<0.001
Age (years)	54.55 ± 12.77	51.70 ± 12.28	64.01 ± 9.41	<0.001
Body mass index (kg/m^2^)	23.41 ± 12.72	23.58 ± 14.38	22.83 ± 3.55	0.601
Urea nitrogen (mmol/L)	6.92 ± 6.70	6.69 ± 7.21	7.69 ± 4.56	0.187
Creatinine (μmol/L)	70.93 ± 39.41	67.66 ± 38.74	81.79 ± 39.88	0.001
Total cholesterol (mmol/L)	3.73 ± 11.24	3.25 ± 1.04	5.32 ± 23.29	0.102
Triglyceride (mmol/L)	0.88 ± 0.61	0.89 ± 0.66	0.84 ± 0.44	0.469
HDL-C (mmol/L)	0.90 ± 0.35	0.92 ± 0.35	0.83 ± 0.34	0.034
LDL-C (mmol/L)	1.77 ± 0.68	1.80 ± 0.67	1.67 ± 0.72	0.096
Alanine aminotransferase (U/L)	26.91 ± 38.35	28.11 ± 42.46	22.91 ± 18.82	0.231
Aspartate aminotransferase (U/L)	37.11 ± 39.40	37.97 ± 43.83	34.24 ± 18.02	0.402
Total bilirubin (μmol/L)	27.87 ± 30.96	27.90 ± 33.40	27.75 ± 21.10	0.966
Alkaline phosphatase (U/L)	92.64 ± 51.21	90.87 ± 49.70	98.48 ± 55.76	0.189
Gamma-glutamyl transferase (U/L)	53.83 ± 62.52	56.02 ± 63.55	46.58 ± 58.68	0.181
Total bile acid (μmol/L)	41.22 ± 56.64	36.95 ± 45.47	55.38 ± 82.35	0.004
Cholinesterase (U/L)	143.47 ± 69.61	150.62 ± 70.82	119.78 ± 59.91	<0.001
Lactate dehydrogenase (U/L)	203.08 ± 80.92	199.37 ± 82.20	215.36 ± 75.61	0.080
Prothrombin time (ser)	14.77 ± 3.14	14.61 ± 2.80	15.30 ± 4.03	0.049
International normalized ratio	1.34 ± 0.28	1.32 ± 0.26	1.38 ± 0.33	0.102
APTT (ser)	34.71 ± 13.73	34.83 ± 15.42	34.34 ± 5.10	0.756
Fibrinogen (g/L)	2.17 ± 1.04	2.22 ± 1.11	2.00 ± 0.77	0.062
PNI	40.01 ± 6.00	41.11 ± 5.73	36.37 ± 5.41	<0.001
Hemoglobin (g/L)	91.47 ± 26.79	93.42 ± 27.90	85.01 ± 21.62	0.005
Platelet count (×10^9^ /L)	94.14 ± 83.14	98.12 ± 88.46	80.96 ± 60.85	0.068
White blood cell count (×10^9^ /L)	3.78 ± 2.89	3.83 ± 2.87	3.59 ± 2.96	0.457
Albumin/Globulins	1.47 ± 1.66	1.52 ± 1.88	1.30 ± 0.39	0.234
MELD score	10.89 ± 4.47	10.66 ± 4.40	11.65 ± 4.62	0.050
CTP score	6.90 ± 1.64	6.70 ± 1.53	7.60 ± 1.80	<0.001
Gender (%)				0.365
Male	284 (64.55%)	222 (65.68%)	62 (60.78%)	
Female	156 (35.45%)	116 (34.32%)	40 (39.22%)	
Smoking (%)				0.375
Yes	313 (71.14%)	244 (72.19%)	69 (67.65%)	
No	127 (28.86%)	94 (27.81%)	33 (32.35%)	
Drink (%)				0.709
Yes	304 (69.09%)	232 (68.64%)	72 (70.59%)	
No	136 (30.91%)	106 (31.36%)	30 (29.41%)	
Diabetes (%)				0.150
Yes	350 (79.55%)	274 (81.07%)	76 (74.51%)	
No	90 (20.45%)	64 (18.93%)	26 (25.49%)	
Hypertension (%)				0.053
Yes	353 (80.23%)	278 (82.25%)	75 (73.53%)	
No	87 (19.77%)	60 (17.75%)	27 (26.47%)	
Etiology (%)				0.003
Viral hepatica	205 (46.59%)	165 (48.82%)	40 (39.22%)	
Alcoholic hepatitis	64 (14.55%)	44 (13.02%)	20 (19.61%)	
Autoimmune hepatitis	25 (5.68%)	13 (3.85%)	12 (11.76%)	
Portal vein cavernous degeneration	17 (3.86%)	17 (5.03%)	0 (0.00%)	
Others	129 (29.32%)	99 (29.29%)	30 (29.41%)	
Surgical indications (%)				<0.001
Gastrointestinal bleeding	271 (61.59%)	222 (65.68%)	49 (48.04%)	
Refractory ascites	139 (31.59%)	88 (26.04%)	51 (50.00%)	
Others	30 (6.82%)	28 (8.28%)	2 (1.96%)	
Previous history of HE (%)				<0.001
Yes	19 (4.32%)	4 (1.18%)	15 (14.71%)	
No	421 (95.68%)	334 (98.82%)	87 (85.29%)	

HDL-C, high-density lipoprotein-cholesterol; LDL-C, low-density lipoprotein-cholesterol; APTT, activated partial thromboplastin time; PNI, prognostic nutritional index; MELD, model for end-stage liver disease; CTP, Child-Turcotte-Pugh; HE, hepatic encephalopathy.

### Variable selection

**Figure 2:**
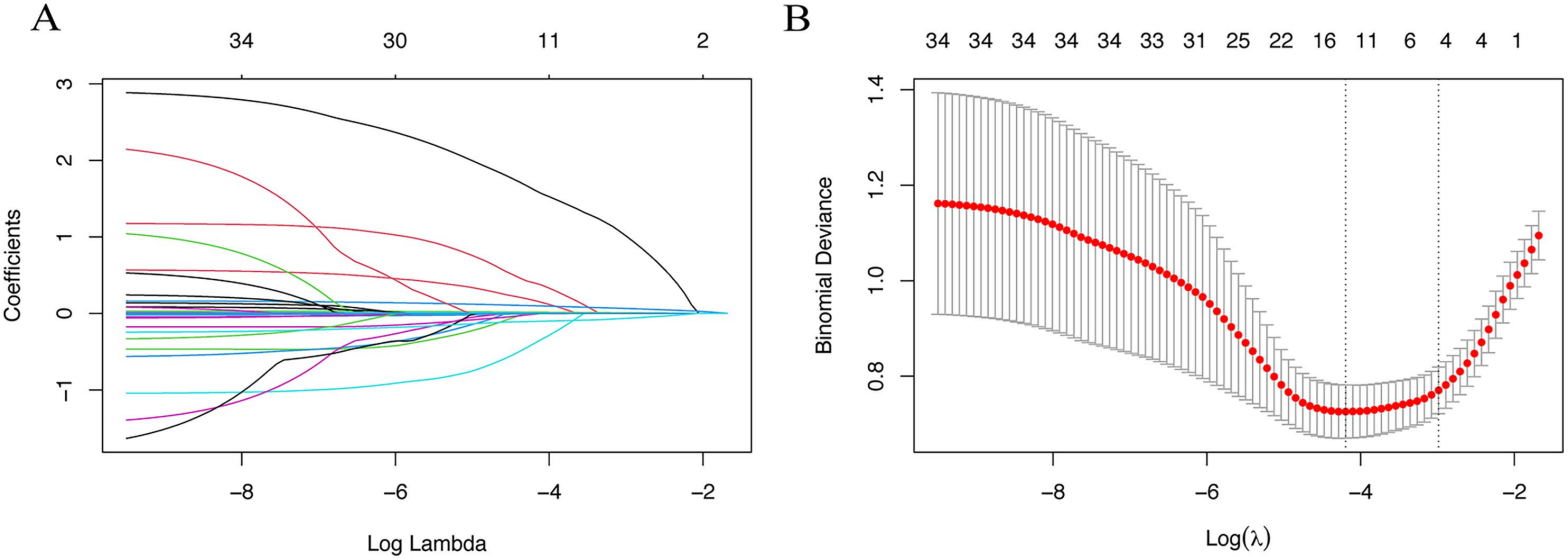
Lasso regression was used to screen predictors of overt hepatic encephalopathy within 1 year after TIPS in patients with cirrhosis. **(A)** Regression coefficients change curves versus log Lambda. **(B)** The optimal *λ* process was obtained by iterative analysis using 10-fold cross-validation.

**Figure 3:**
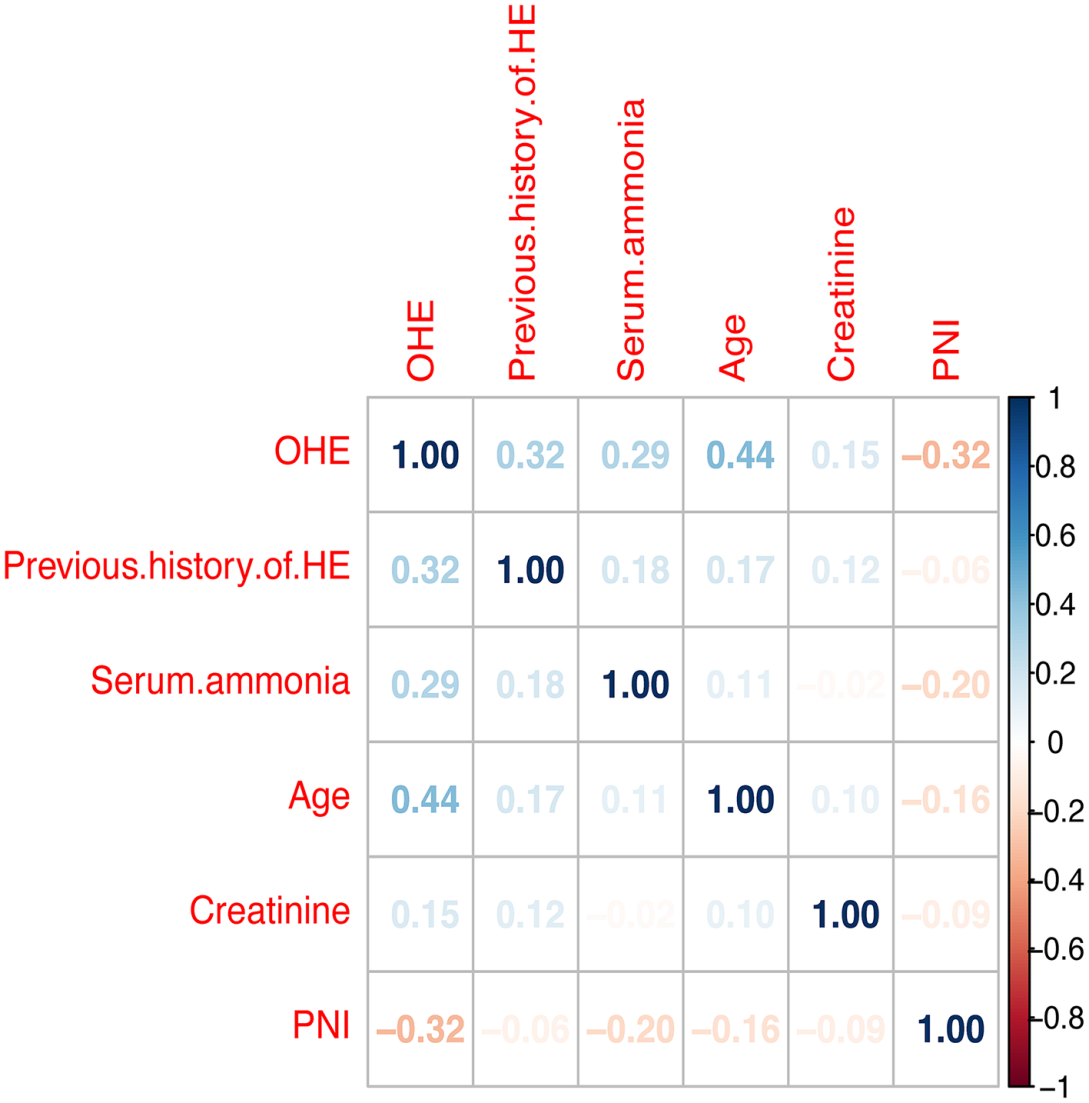
Pearson’s analysis of the occurrence of overt hepatic encephalopathy in cirrhotic patients 1 year after TIPS and its influencing factors. HE, Hepatic Encephalopathy; OHE, Overt Hepatic Encephalopathy; PNI, Prognostic nutritional index.

To reduce complexity and overfitting, 34 candidate variables were analyzed by LASSO regression (Figure 2A). Using 10-fold cross-validation (Figure 2B), *λ*.1se = 0.025 was selected, retaining five predictors: PNI, age, prior HE, ammonia, and creatinine. Spearman analysis (Figure 3) showed age had the strongest positive correlation with OHE, while creatinine was weakest. PNI showed a protective effect—higher PNI correlated with lower OHE risk.

### Model construction

**Figure 4:**
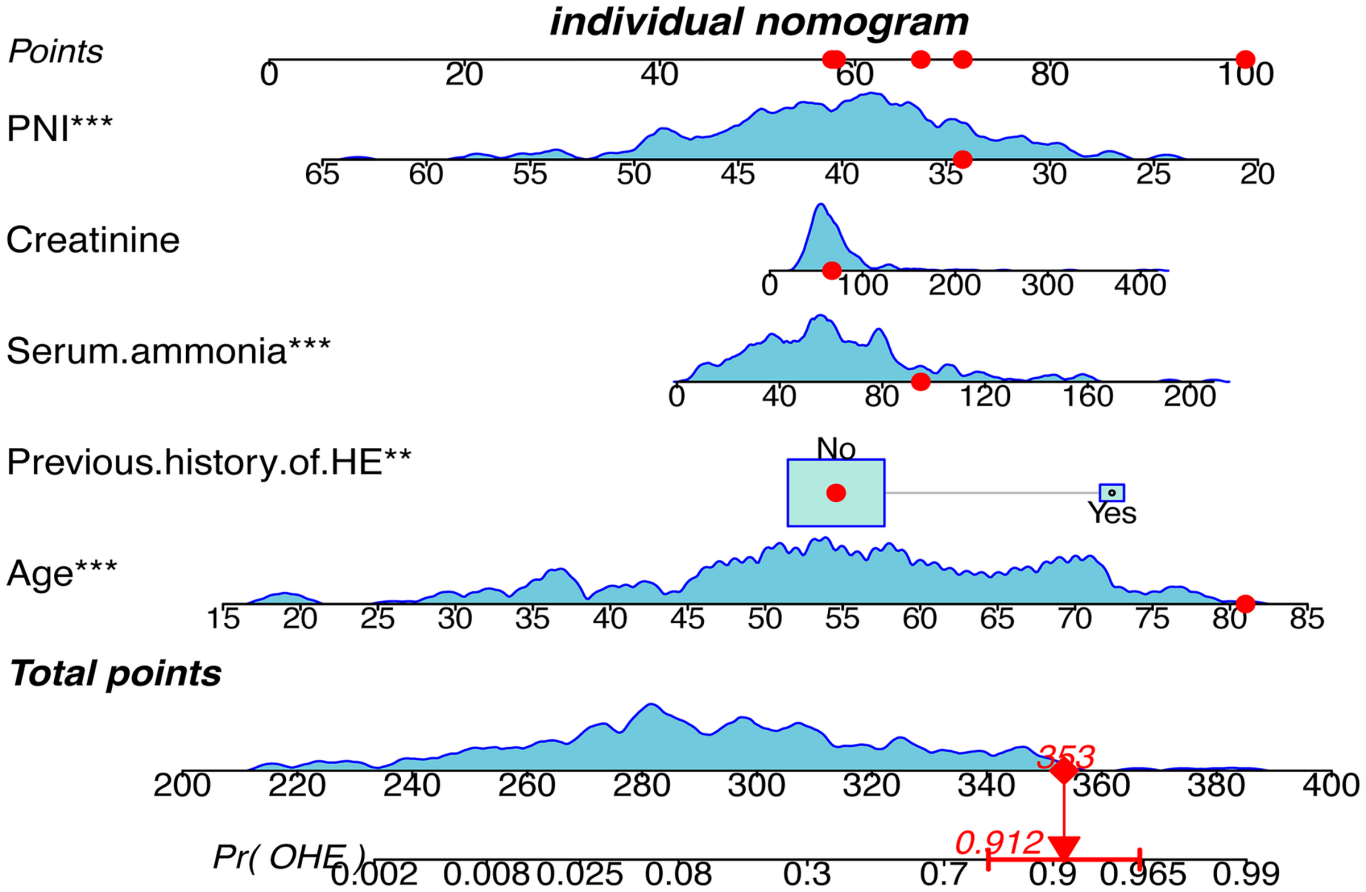
Nomogram depicting the clinical prediction model for the occurrence of OHE within 1 year after TIPS in patients with cirrhosis. HE, Hepatic Encephalopathy; OHE, Overt Hepatic Encephalopathy; PNI, Prognostic nutritional index.

A multivariate logistic regression model was constructed using the five variables, and a nomogram was drawn (Figure 4). Each predictor was assigned points, and total score mapped to 1-year OHE probability. For example, a score of 353 corresponded to a 91.2% risk (95% CI: 78.2–96.1%).

### Model validation and comparison

**Figure 5:**
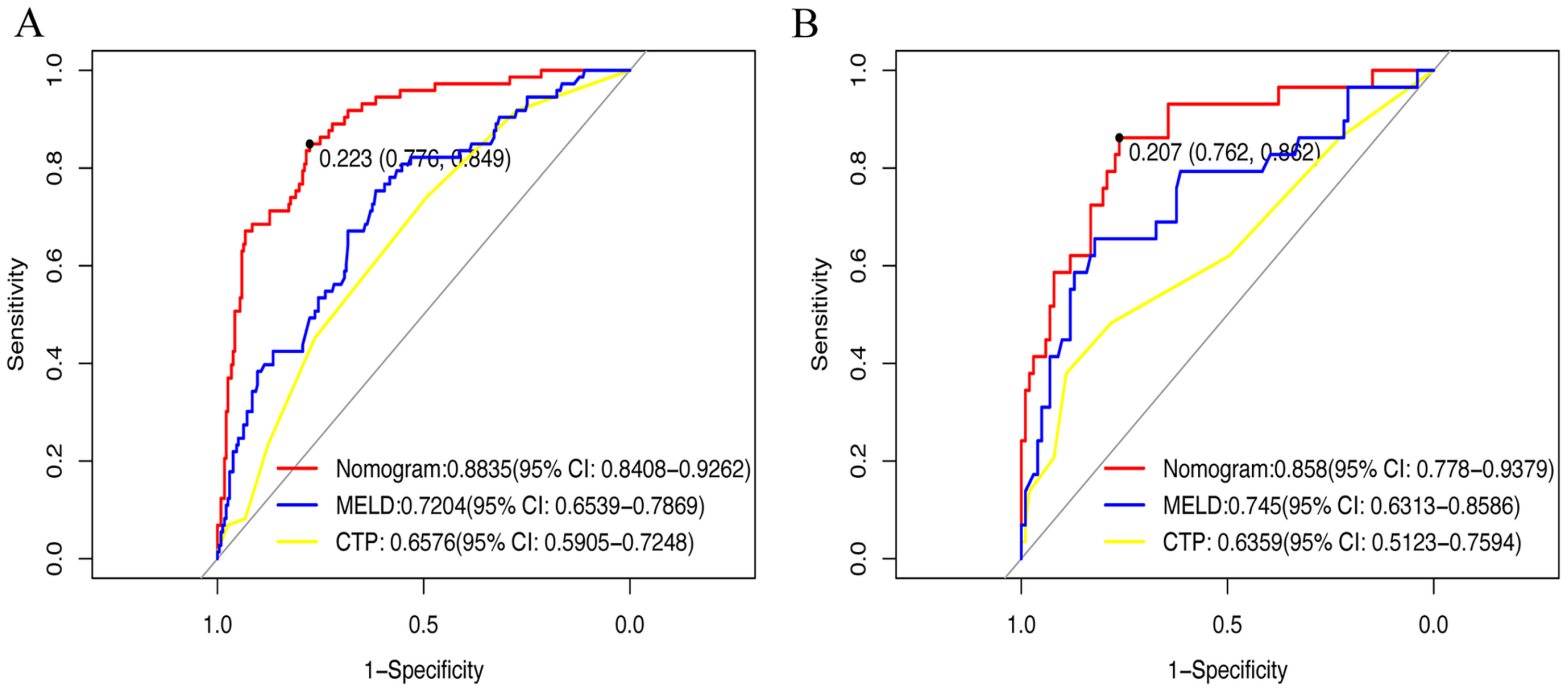
Receiver operating characteristic curve was used in clinical prediction models. **(A)** Training set; **(B)** Validation set. CTP, Child-Turcotte-Pugh; MELD: Model for End-Stage Liver Disease.

**Figure 6:**
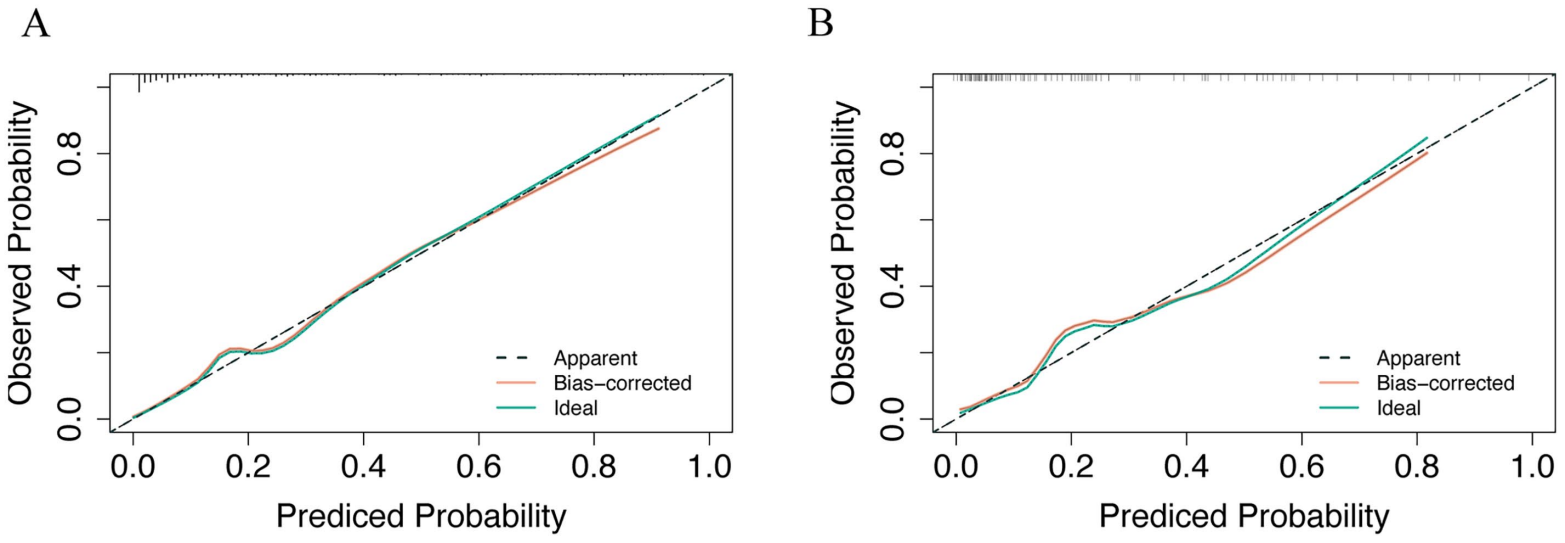
Calibration curve was used in clinical prediction models. **(A)** Training set; **(B)** Validation set.

ROC curves (Figures 5A,B) demonstrated superior performance of the model vs. MELD and CTP. In the training cohort, AUC = 0.8835 (95% CI: 0.745–0.836) vs. MELD (0.7204) and CTP (0.6576). In the validation cohort, AUC = 0.858 vs. MELD (0.745) and CTP (0.6359). DeLong’s test was performed to assess the statistical significance of differences between paired ROC curves, and the results are presented in Supplementary Table S1. The nomogram showed significantly higher discrimination than MELD and CTP, whereas the difference between MELD and CTP was not statistically significant. Calibration curves (Figures 6A,B) showed high agreement between predicted and observed probabilities.

### Model evaluation

**Figure 7:**
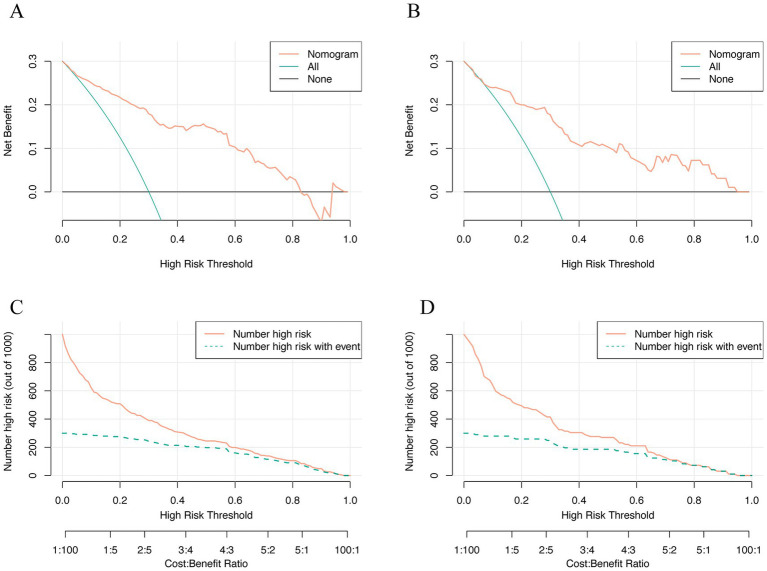
Decision curve analysis and clinical impact curve were used in clinical prediction models. **(A)** Training set; **(B)** Validation set; **(C)** Training set; **(D)** Validation set.

DCA (Figures 7A,B) demonstrated that the nomogram consistently outperformed the “treat all” and “treat none” strategies across the evaluated risk-threshold range (0.1–0.8). Within this interval, the net benefit values achieved by the model ranged approximately from 0 to 0.25. CIC (Figures 7C,D) indicated that above 60% threshold, the predicted high-risk population closely matched actual OHE cases, minimizing error and confirming clinical utility.

## Discussion

In this single-center retrospective cohort of 440 patients undergoing first-time TIPS, we developed and internally validated a nomogram incorporating five preoperative variables (PNI, age, prior HE, serum ammonia, and creatinine) to predict the 1-year risk of OHE. The nomogram demonstrated good discrimination (AUC 0.88 training; 0.86 validation), favorable calibration, and superior clinical net benefit compared with MELD and CTP scoring systems.

These findings are clinically meaningful because, although TIPS effectively relieves portal hypertension and reduces complications such as variceal bleeding and refractory ascites, post-TIPS OHE remains a major challenge that compromises quality of life, increases healthcare costs, and may necessitate shunt reduction (15). Given the limited predictive tools for post-TIPS OHE, a simple, accurate model based on preoperative data is essential. Nomograms provide a user-friendly visual representation of complex models, integrating multiple predictors to estimate individualized risk, and are increasingly applied in clinical practice (16). This study identified five significant predictors—PNI, age, prior HE, serum ammonia, and creatinine—using LASSO regression. Elevated age, ammonia, creatinine, and prior HE were associated with increased OHE risk, consistent with previous studies (4, 17–20). Nevertheless, previous models for predicting post-TIPS OHE have primarily relied on conventional scores such as MELD, CTP, or single laboratory markers (e.g., ammonia). However, their discriminatory ability and clinical applicability have been limited. Our study extends this body of work by incorporating both traditional risk factors and the nutritional-inflammatory marker PNI, thereby improving predictive accuracy and providing a more practical tool for individualized risk stratification. Importantly, our findings additionally highlight nutritional and immune status, as reflected by PNI, as an independent protective factor, suggesting that good preoperative nutritional status may reduce OHE risk.

HE is a neuropsychiatric syndrome caused by liver dysfunction and portosystemic shunting, primarily driven by hyperammonemia (21). When liver detoxification is impaired (e.g., after TIPS), ammonia enters the systemic circulation, leading to cognitive impairment (22–24).

The present study also found that elevated serum creatinine levels were associated with the occurrence of OHE in TIPS in cirrhotic patients. Creatinine level is indicative of renal function, and research has identified renal insufficiency as a significant risk factor for HE (25, 26). In the presence of impaired hepatic function, impaired urea cycling limits hepatic clearance of ammonia, and the kidney becomes a key organ for compensatory ammonia excretion. As renal function deteriorates, renal tubular ammonia secretion is reduced, thereby exacerbating hyperammonemia. This suggests an important role for liver-kidney axis in ammonia metabolism.

A highlight of this study is incorporating the nutritional/inflammatory composite index PNI into post-TIPS OHE prediction. PNI (albumin + lymphocyte count) was originally used to assess nutritional status in gastric cancer (27) and has since been applied to cervical cancer (28), diabetic nephropathy (29), and cognitive decline in the elderly (30). Low PNI indicates malnutrition and immune compromise. Malnutrition elevates systemic inflammation, increasing HE risk (31). In recent years, the role of inflammation in the pathogenesis of HE has attracted increasing attention. Interleukin-6 (IL-6), a multifunctional cytokine involved in immune regulation, metabolic control, inflammatory response, and neuroregulation, has been shown to be closely associated with the development of HE (32–34). Elevated IL-6 levels in cirrhotic patients have been linked to a significantly increased risk of OHE following TIPS, underscoring the potential value of IL-6 as a biomarker for predicting post-TIPS HE risk (33). The interaction between nutritional and inflammatory status plays a critical role in the development of HE in patients after TIPS.

The relationship between nutritional status and HE has also become a research focus in recent years. Malnutrition-related sarcopenia may contribute to the pathogenesis of OHE by impairing muscle-based ammonia metabolism. In cirrhotic patients, skeletal muscle acts as an important extrahepatic route for ammonia detoxification through glutamine synthetase activity (35, 36). Loss of muscle mass compromises this compensatory pathway, leading to elevated blood ammonia levels. Furthermore, hyperammonemia itself can induce the expression of myostatin, a muscle growth inhibitor, thereby exacerbating sarcopenia and creating a vicious cycle. In this study, the inclusion of the PNI in the prediction model provides a simple, cost-effective, and practical surrogate for assessing preoperative nutritional and immunoinflammatory status, offering valuable guidance for clinical decision-making.

In this study, the model not only demonstrated strong discrimination and calibration in the training cohort but also maintained high performance in the independent validation cohort. Compared to traditional scores (MELD, CTP), the model significantly improved predictive accuracy and clinical utility. DCA and CIC confirmed that applying this model could provide greater net clinical benefit, aiding in early identification of high-risk patients and timely intervention, thus improving prognosis and reducing OHE-related healthcare burden. Nevertheless, several limitations should be acknowledged. First, as a single-center retrospective study, selection and information biases cannot be fully excluded. Second, although internal validation was performed, external validation using multicenter, prospective cohorts is needed to confirm the generalizability of the model. Additionally, our model relies solely on preoperative baseline variables, which may not capture dynamic changes in OHE risk over time. Future studies incorporating longitudinal data and emerging biomarkers could further enhance predictive accuracy and clinical applicability.

## Conclusion

In conclusion, the present study developed and validated a predictive model to estimate the 1-year risk of OHE in patients with cirrhosis after TIPS. By providing an intuitive nomogram tool, our model can help clinicians make informed decisions regarding patient selection, preoperative optimization, and early postoperative management.

## Data Availability

The raw data supporting the conclusions of this article will be made available by the authors, without undue reservation.
