# Supplementary material for: Development and validation of a preoperative clinical parameter-based nomogram to predict overt hepatic encephalopathy within 1 year after transjugular intrahepatic portosystemic shunt
Source: Front Med (Lausanne). 2025 Oct 20;12:1634368. doi: 10.3389/fmed.2025.1634368 (PMC12580194; doi:10.3389/fmed.2025.1634368)
Supplement: Supplementary file 1 [file Table_1.DOCX]

| **Comparison** | **AUC of Model 1** | **AUC of Model 2** | **Z value** | **95% CI for AUC difference** | **p-value** |
| --- | --- | --- | --- | --- | --- |
| Nomogram vs MELD | 0.884 | 0.720 | 5.61 | 0.112 – 0.232 | <0.001 |
| Nomogram vs CTP | 0.884 | 0.658 | 7.18 | 0.170 – 0.298 | <0.001 |
| MELD vs CTP | 0.720 | 0.658 | 1.93 | -0.001 – 0.127 | 0.054 |

**Supplementary Table S1. Comparison of predictive performance between the nomogram model, MELD and CTP score.**

Abbreviation: AUC: area under the ROC curve; MELD: Model for End-stage Liver Disease; CTP: Child-Turcotte-Pugh.
